# Osmotic stress tolerance and transcriptome analysis of *Gluconobacter oxydans* to extra-high titers of glucose

**DOI:** 10.3389/fmicb.2022.977024

**Published:** 2022-08-12

**Authors:** Xinlu Liu, Zhiwei Wang, Jianjian Xiao, Xin Zhou, Yong Xu

**Affiliations:** ^1^Key Laboratory of Forestry Genetics and Biotechnology, Ministry of Education, Nanjing Forestry University, Nanjing, China; ^2^Jiangsu Co-Innovation Center of Efficient Processing and Utilization of Forest Resources, College of Chemical Engineering, Nanjing Forestry University, Nanjing, China; ^3^Jiangsu Province Key Laboratory of Green Biomass-based Fuels and Chemicals, Nanjing, China

**Keywords:** *Gluconobacter oxydans*, extra-high titers of glucose, lignocellulosic sugar fermentation, osmotic stress tolerance, transcriptome analysis

## Abstract

*Gluconobacter oxydans* has been widely acknowledged as an ideal strain for industrial bio-oxidations with fantastic yield and productivity. Even 600 g/L xylose can be catalyzed efficiently in a sealed and compressed oxygen-supplying bioreactor. Therefore, the present study seeks to explore the osmotic stress tolerance against extra-high titer of representative lignocellulosic sugars like glucose. *Gluconobacter oxydans* can well adapted and fermented with initial 600 g/L glucose, exhibiting the highest bio-tolerance in prokaryotic strains and the comparability to the eukaryotic strain of *Saccharomyces cerevisiae*. 1,432 differentially expressed genes corresponding to osmotic pressure are detected through transcriptome analysis, involving several genes related to the probable compatible solutes (trehalose and arginine). *Gluconobacter oxydans* obtains more energy by enhancing the substrate-level phosphorylation, resulting in the increased glucose consumption rate after fermentation adaption phase. This study will provide insights into further investigation of biological tolerance and response to extra-high titers of glucose of *G. oxydans*.

## Introduction

*Gluconobacter oxydans* (*G. oxydans*), an obligate aerobic Gram-negative bacterium belonging to the family *Acetobacteraceae*, is common in the sugar-enriched environments, such as nectars, flowers, fruits, and fermented foods (Raspor and Goranovič, 2008; Qin et al., 2021). Its respiratory metabolism is characterized by rapid selective oxidation of oxy-compounds to obtain energy from incomplete oxidation (Ehrenreich and Liebl, 2017; Lynch et al., 2019). The catalytic reaction center of the membrane-bound dehydrogenases participating in the incomplete oxidation is directed toward the periplasmic space, making substrate and product transport through cell membrane unnecessary (Deppenmeier et al., 2002; Keliang and Dongzhi, 2006; Gao et al., 2020) and rapid accumulation of incompletely oxidized product in the medium (Keliang and Dongzhi, 2006; Zhou et al., 2018).

So far, this bacterial strain has been adopted for the bioconversion of various aldonic acids (Jin et al., 2019), hydroxy acids (Hua et al., 2019), ketones (Chen et al., 2021), and furan acids (Du et al., 2021), etc., using a sealed and compressed oxygen supply biotechnology (SOS) with whole-cell catalysis (Hua et al., 2020b). Hua et al. obtained ultrahigh-titer erythrulose at 364.7 g/L by SOS technology and exceeded the inhibitory concentration of erythrulose (about 250 g/L; Hua et al., 2020b). Even 600 g/L xylose was fermented efficiently at the productivity of 4.69 g/L/h (Zhou et al., 2015). Therefore, *G. oxydans* has gained worldwide attention lately as an ideal strain for industrial bio-oxidation.

According to previous knowledge, *G. oxydans* often encounters dehydration in a hypertonic medium, impeding the cellular metabolism, fermentation, growth, and survival (Zahid et al., 2015). Microorganisms have developed two basic survival strategies, the “salt in” and the “compatible solutes” to resist osmotic stress generated by decreased water activity (Vyrides and Stuckey, 2017). For instance, anaerobic halophilic bacteria counteract osmotic stress by the first strategy and allow the intracellular enzymes to adapt to the newly elevated ion titer environment (Naufal and Wu, 2020). In this regard, many microorganisms often apply the compatible solute strategy without special adaptation, which are the uncharged, polar, and hydrosoluble compounds at physiological pH conditions (Brown and Simpson, 1972). Few compounds, meeting the specific requirements, could be classified into 4 structural categories: sugars, polyols, free amino acids and its derivatives, and heterosides (Vyrides and Stuckey, 2017; Liang et al., 2020; Gregory and Boyd, 2021). So far, only mannitol has been identified as a compatible solute in *G. oxydans* against the sucrose-mediated osmotic pressure (Zahid et al., 2015).

Herein, we studied the effect of the extremely high titer of main lignocellulosic sugar of glucose on the cellular metabolism, fermentation, growth, and survival of *G. oxydans*. High-throughput transcriptome sequencing technology was used to generate transcriptional profiles of *G. oxydans* against sugar osmotic stress. The comparative study of these profiles might reveal the genes responsible for osmotic tolerance and help to understand the molecular mechanisms associated with the hyperosmotic stress tolerance of *G. oxydans*.

## Materials and methods

### Strains and growth conditions

*Gluconobacter oxydans* NL71 strain (derived from ATCC 621H) was cultured in a yeast extract (0.5%)-sorbitol (5%) medium (YS) and incubated at 30°C and 220 rpm for 24 h. *Escherichia coli* (*E. coli*) was cultured in an LB medium and incubated at 37°C and 200 rpm for 12 h. *Saccharomyces cerevisiae* (*S. cerevisiae*) was cultured in a yeast extract (0.3%)-peptone (0.5%)-glucose (2.0%) medium (YPG) and incubated at 30°C and 150 rpm for 24 h.

#### Cultural conditions

The synthetic medium (g/L) contained yeast extract (5.0), (NH_4_)_2_SO_4_ (5.0), K_2_HPO_4_ (2.0), KH_2_PO_4_ (1.0), MgSO_4_ (0.5), ZnCl_2_(0.4), CaCl_2_(0.2), and different titers of glucose (100, 200, 400, or 600).

Fermentations were performed in 250 ml Erlenmeyer flask containing 50 ml medium for 24–48 h shaking at optimum temperature and speed for each strain. The initial OD_600_ for fermentation was 2.0. The pH value of medium containing *G. oxydans* was stabilized between 5 and 6 using 50% NaOH solution.

#### Spot assay

*Saccharomyces cerevisiae*, *E. coli*, and *G. oxydans* cells in logarithmic growth phase were diluted with saline to an absorbance at 600 nm (OD_600_) of 2.0. 4 μl of 10-fold serial dilutions were spotted on YS, LB, or YPG-agar plates containing different titers of glucose. Cell growth was evaluated after 36 h of incubation at the optimum temperature for each strain.

#### CFU counts

To analyze cell viability, appropriate dilutions of *G. oxydans* in synthetic medium containing different titers of glucose were plated onto YS-agar plates at various time points. The YS-agar plate contained yeast extract (0.5%), sorbitol (5%), and agar (1.5%). Colony-forming units (CFU) were calculated by counting the number of viable colonies after 2 days of incubation at 30°C.

#### Whole-cell activity assays

Whole-cell activity assays were analyzed by a method developed by Peters et al. (2017). Briefly, cells of *G. oxydans* obtained at different time points from different titers of glucose were harvested and washed twice with normal saline and were resuspended in phosphate buffer (pH 5.5). The whole-cell 2,6-dichlorophenolindophenol (DCPIP) assays were performed in 96-well plates (Thermo Fisher Scientific, America). Each well contained 166 μM of DCPIP and 110 μM of phenazine methosulfate, combined with cells (OD_600_ of 0.2) and 25 mM glucose. The reaction was measured by Infinite 200 PRO Tecan microplate readers (TECAN, Switzerland) at 594 nm for 30 cycles at 30°C and was shaken every 10 s. The oxidative activity of the substrate is quantified by linear regression of the initial absorbance reduction. One unit of oxidation activity was defined as 1 μmol glucose oxidized per minute as determined by a reduction of 1 μmol DCPIP (Peters et al., 2017). The extinction coefficient of DCPIP at 594 nm was 5,800 L/(mol·cm) with a pH of 5.5.

#### Determination of ATP

Cells obtained at different time points from different titers of glucose were harvested and washed twice with normal saline and were resuspended in phosphate buffer. The determination of adenosine triphosphate (ATP) was carried out by ATP Assay Kit (Beyotime Biotechnology, China) according to the manufacturer’s protocol. The samples were placed in 96-well white plates (Corning Incorporated, America) and their chemiluminescence values were measured using an Infinite 200 PRO Tecan microplate readers (TECAN, Switzerland). The protein content was determined by the Bradford Protein Assay Kit (Beyotime Biotechnology, China) according to the manufacturer’s protocol.

#### RNA-seq analysis

The *G. oxydans* was cultured to logarithmic phase and then reinoculated into the synthetic medium at 100, 400, and 600 g/L glucose at an initial OD_600_ of 2.0. After incubation for 8 h, cells were harvested and washed twice with normal saline. Total RNA was isolated using the Bacterial RNA Miniprep Kit (Biomiga, America) and stored at −80°C. The concentration and quality of total RNA were determined by NanoPhotometer spectrophotometry (IMPLEN, America) and Agilent 2100 Bioanalyzer (Agilent Technologies, America), respectively. The frozen samples were shipped to Gene Denovo Biotechnology Co. Ltd. (Guangzhou, China) for RNA-seq analysis using the Illumina NovaSeq 6000 platform (Liu et al., 2017, 2021). The raw sequencing data in this study have been submitted to the NCBI SRA database under the BioProject number of PRJNA755830. A parallel experiment was conducted in triplicate.

#### qRT-PCR

RNA was extracted as described in the section RNA-Seq analysis. Real-time quantitative polymerase chain reaction (qRT-PCR) was performed using SYBR Premix ExTaq Kit (TaKaRa, Japan) in ABI StepOnePlus^TM^ Real-Time PCR System (Applied Biosystems, America), as reported previously (Miao et al., 2017). The primers for qRT-PCR were listed in Table 1. Relative gene expression was calculated using the 2^−ΔΔCt^ method with 16S rRNA as internal control. A parallel experiment was conducted in triplicate.

**Table 1:** Primers used for qRT-PCR in this study.

Gene	Sequence (5′–3′)
16S-F	AGGACCTGATTACTGTCTTCGG
16S-R	TTCCACGCACCATTTCTTC
VZ55_RS06850-F	GTTGGACAGGCTGTTGCG
VZ55_RS06850-R	TAGGGGGAGATATTCGGG
VZ55_RS10065-F	GAACCTCGTTTACATCCCA
VZ55_RS10065-R	GACAGCTTACCCGTCTCTG
VZ55_RS08395-F	CGGTGAGATTGAGTGGGC
VZ55_RS08395-R	ATCGGCAGGTAAAGCAGG
VZ55_RS05735-F	TGGACAGCACCAAGAGCA
VZ55_RS05735-R	CCCGAGAGGAGCATCAGA
VZ55_RS08235-F	ACACCGACCAACAAACCT
VZ55_RS08235-R	ATTCCAAGCGGGACGTAA

F/R: forward primer/reverse primer.

#### Intracellular arginine

The *G. oxydans* was cultured to logarithmic phase and then reinoculated into the synthetic medium at 200, 400, and 600 g/L glucose at an initial OD_600_ of 2.0. After incubation for 8 h, cells were harvested and freeze-dried. The intracellular substances were extracted using the method of Zahid et al. (2015). The extract was added with sulfosalicylic acid (final concentration 4%) at 4°C overnight and freeze-dried. After freeze-dried sample was dissolved, the arginine concentration was determined by automatic amino acid analyzer (Sykam, Germany).

#### Analytical methods

Glucose, sodium gluconate, 2-keto-gluconic acid hemicalcium salt hydrate, and 5-keto-gluconic acid potassium salt hydrate were all purchased from Sigma.

Glucose, glucose acid (GA), 2-keto-D-gluconic acid (2-KGA), and 5-keto-D-gluconic acid (5-KGA) were quantitatively determined by high-performance anion exchange chromatography coupled with pulsed amperometric detection (Dionex ICS-3000), and equipped with CarboPac™ PA200 column with a flow rate of 0.3 ml/min at 30°C according to reports from Zhou et al. (2017).

The data were analyzed by SPSS Statistical Software. T-test was employed for differences between groups. Significant differences for results were considered when *p* < 0.05.

## Results

### The glucose titer effect on the growth and fermentation of *Gluconobacter oxydans* (vs. *Escherichia coli* and *Saccharomyces cerevisiae*)

**Figure 1:**
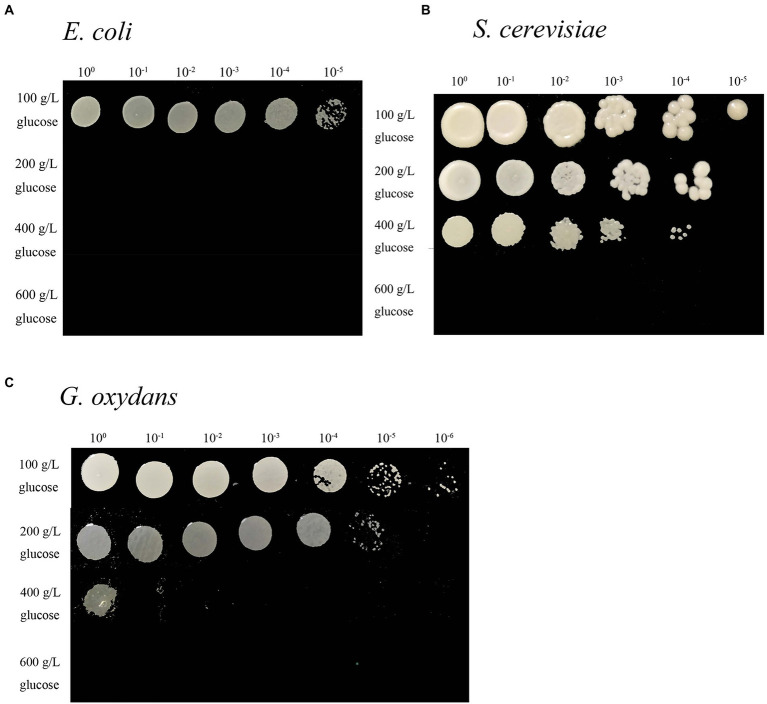
Spot assays of the *E. coli* **(A)** grown on LB medium, *S. cerevisiae* grown on YPG medium **(B)**, and *G. oxydans* **(C)** grown on YS medium at different glucose titers.

The effect of glucose content on microorganism growth was demonstrated by the change in opacity and colony size in the colony pattern (Zhang et al., 2016). In this study, the growth ability of *G. oxydans* with another classical Gram-negative bacterial type strain *Escherichia coli* (*E. coli*) and the eukaryotic type strain *S. cerevisiae* on 100–600 g/L glucose agar plates was compared (Figure 1). *Gluconobacter oxydans* could grow in white and round colonies on a 100 g/L glucose plate. The opacity of colonies decreased with the increase of glucose titers. Only undiluted samples could obtain colonies on 400 g/L glucose agar plate (Figure 1A). The opacity and diameter of *S. cerevisiae* colonies gradually decreased with the increase of glucose content, but it could not grow colonies on an agar plate at 600 g/L glucose content (Figure 1B). *Escherichia coli* could grow on a 100 g/L LB agar plate, but it did not show any growth at 200 g/L glucose content (Figure 1C).

**Figure 2:**
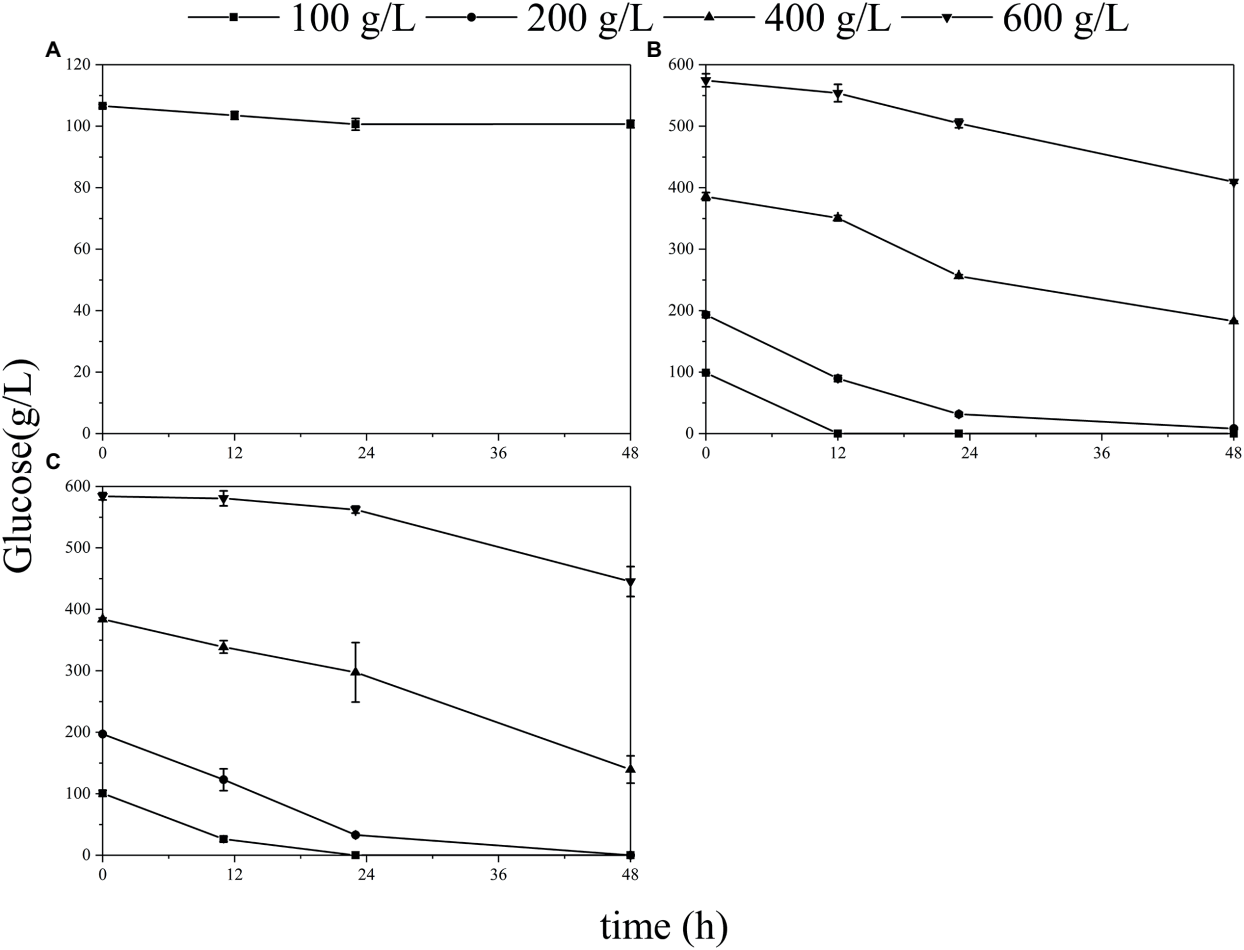
Glucose consumption of the *E. coli* **(A)**, *S. cerevisiae* **(B),** and *G. oxydans* **(C)** at different glucose titers.

Effects of different initial titers of glucose in the liquid medium on the fermentation ability of each microorganism were compared (Figure 2). *Gluconobacter oxydans* and *S. cerevisiae* showed similar trends in the liquid medium. When the initial glucose titer was 100–200 g/L, its consumption rate was rapid within 24 h, reaching 7.56 g/L/h for *G. oxydans* (Figure 2C) and 7.03 g/L/h for *S. cerevisiae* (Figure 2B). Moreover, these two strains could not grow on the 600 g/L glucose agar medium while showing a certain metabolic capacity in the corresponding liquid medium. In contrast, *E. coli* was unable to metabolize at 100 g/L glucose solution (Figure 2A) but grew on the equal concentration of the solid medium.

### The effect of high glucose titers on the physiological status of *Gluconobacter Oxydans*

**Figure 3:**
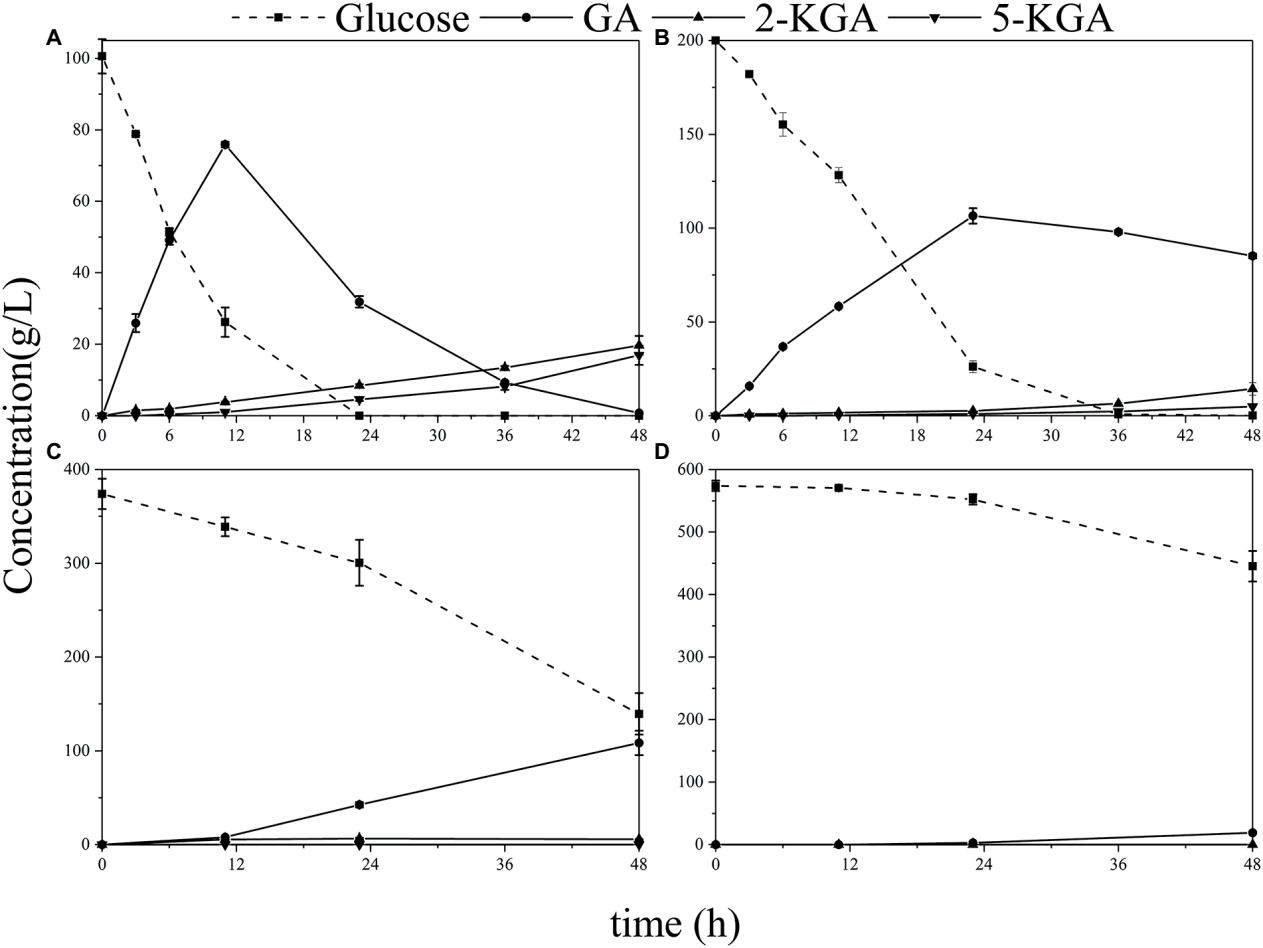
The production of GA, 2-KGA and 5-KGA in various glucose titers. **(A)** Initial 100 g/L glucose; **(B)** initial 200 g/L glucose; **(C)** initial 400 g/L glucose; **(D)** initial 600 g/L glucose.

The fermentation of *G. oxydans* was carried out in the synthesis medium with glucose titers ranging from 100 to 600 g/L. As illustrated in Figure 3A, gluconic acid (GA) was metabolized to 2-keto gluconic acid (2-KGA) and 5-keto gluconic acid (5-KGA) after 18 h, which was consistent with the previous study results (Zhou et al., 2017). When the initial glucose titer was increased to 200 g/L (Figure 3B), glucose was consumed completely within 36 h. When the initial glucose titer was 400 g/L (Figure 3C), there was still 37.3% of glucose remained left in the medium after 48 h of fermentation. *Gluconobacter oxydans* eventually consumed only 22.5% of the glucose in the medium and was incapable of producing keto-gluconic acid with initial 600 g/L glucose (Figure 3D).

**Figure 4:**
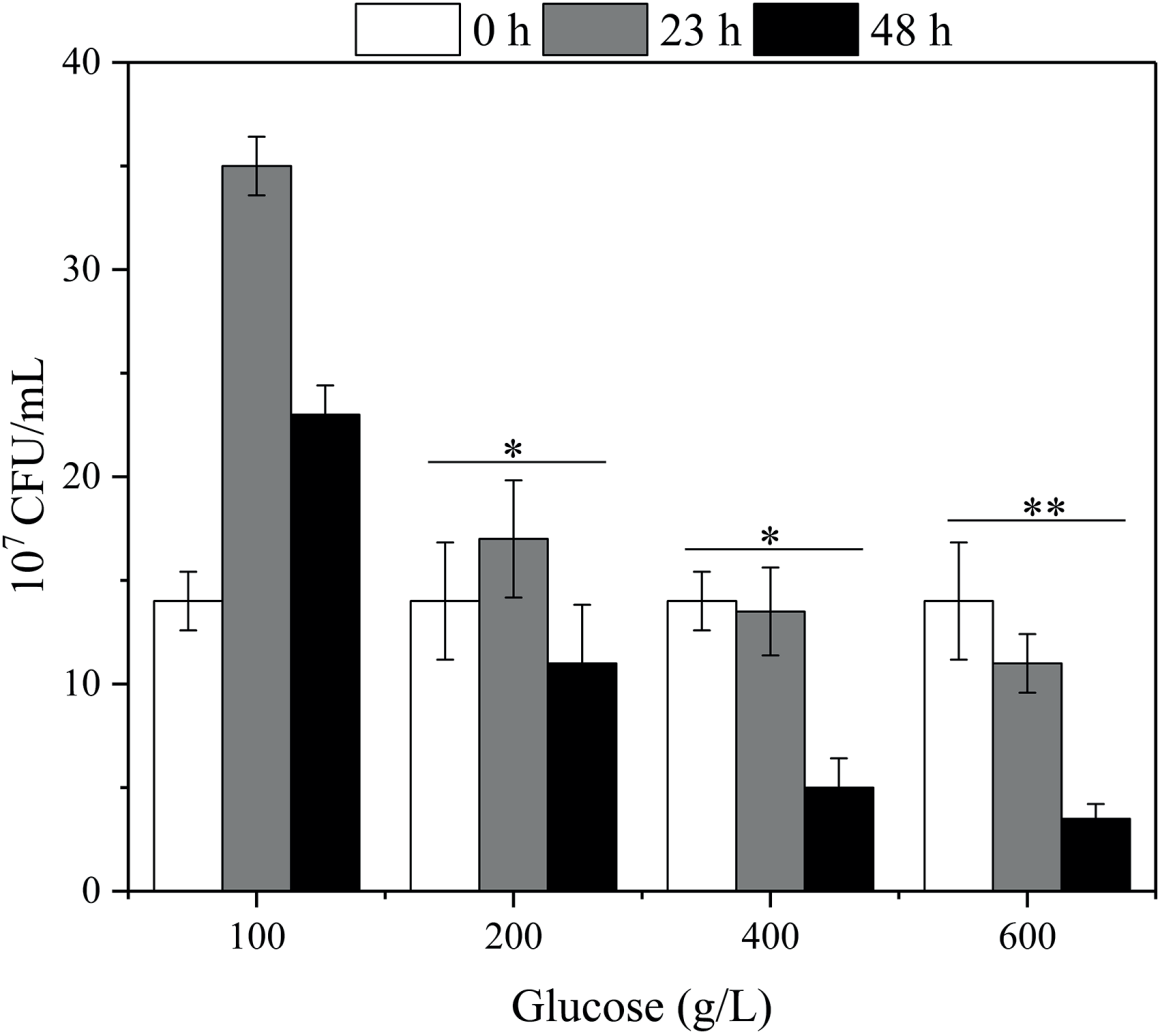
The effect of glucose titers on the viable count of the *G. oxydans* (^*^p < 0.05, ^**^p < 0.01).

Herein, the effect of high glucose titer on the viable count was first analyzed to investigate the effect of high glucose concentration on the physiology and biochemistry of *G. oxydans* during fermentation (Figure 4). At 100 g/L glucose, the viable count increased and then fell due to the proliferation of *G. oxydans* in the presence of glucose. Subsequently, *G. oxydans* began to die and the viable count gradually decreased due to the shortage of glucose. However, the viable count did not change a lot at 200 g/L glucose, indicating that *G. oxydans* could not use glucose to proliferate in this situation. In this process, *G. oxydans* only converted glucose into GA and converted GA to 2/5-KGA when glucose was depleted. When titer was further increased to 400–600 g/L, the viable count decreased by 3.6% and 21% within 24 h and further decreased to 62.9% and 68.2% at 48 h. As illustrated in Figure 3C, when the glucose titer was increased to 400 g/L, the glucose consumption rate reached 6.72 g/L/h during 24–48 h, which was 2.21 times higher than that within 23 h.

**Figure 5:**
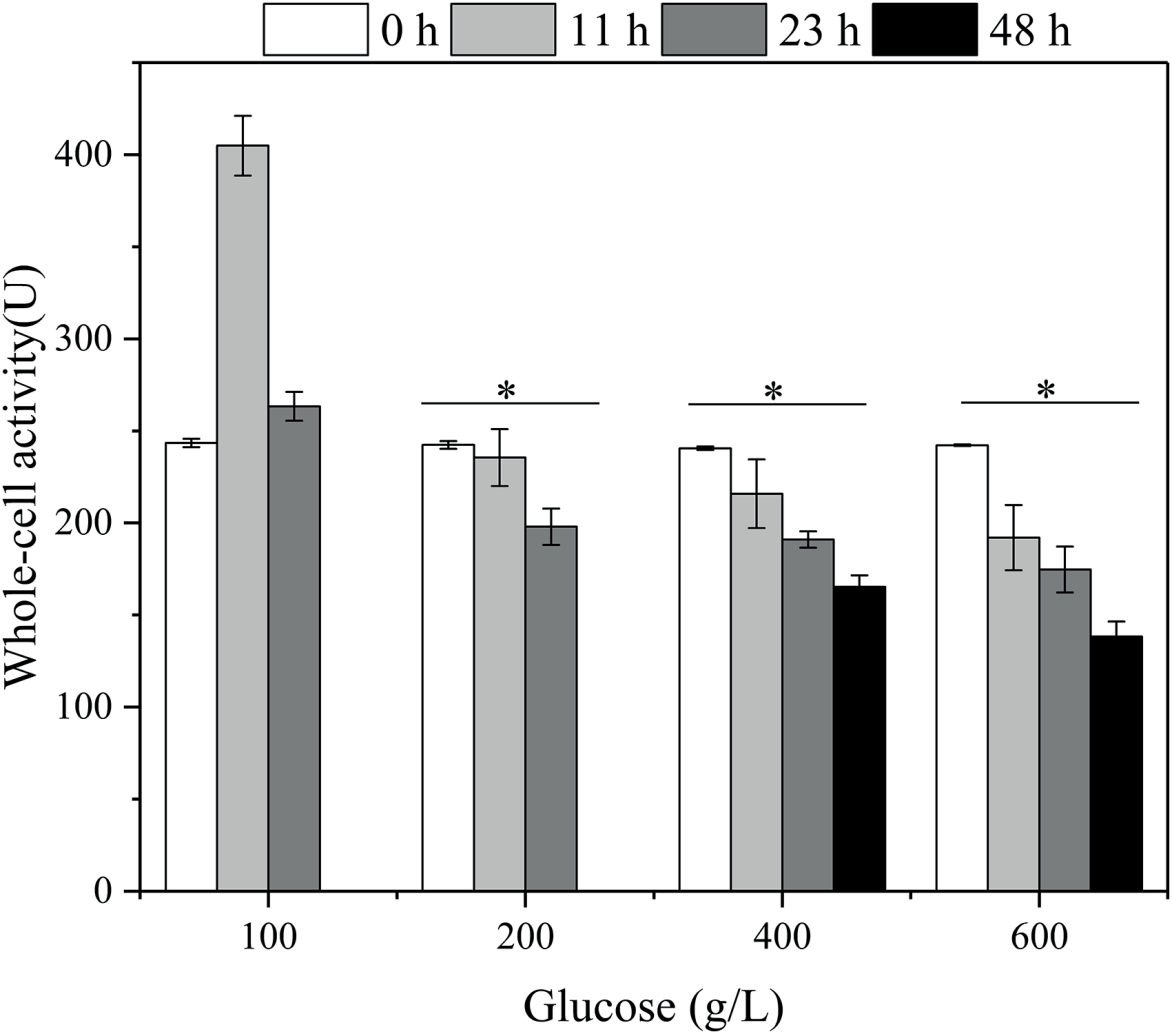
The effect of glucose titers on the whole-cell activity of the *G. oxydans* (^*^p < 0.05).

Besides, the relationship between the whole-cell activity of *G. oxydans*, initial glucose titers, and fermentation time with glucose as substrate was established (Figure 5). The highest activity of *G. oxydans* was 404.9 U at 12 h, which was 1.66 times higher than the original one with initial 100 g/L glucose. Subsequently, the whole-cell activity was reduced to the same level as initially. In combination with the viable count (Figure 4), the membrane-bound enzymes associated with glucose-catalyzed metabolism were coupled to the growth of *G. oxydans* and had the highest activity during their proliferation period. When the initial titer was 200–600 g/L, the whole-cell activity of *G. oxydans* decreased continuously with an increase of fermentation time. The whole-cell activity decreased by 31.3% (400 g/L) and 42.9% (600 g/L) at 48 h, respectively.

**Figure 6:**
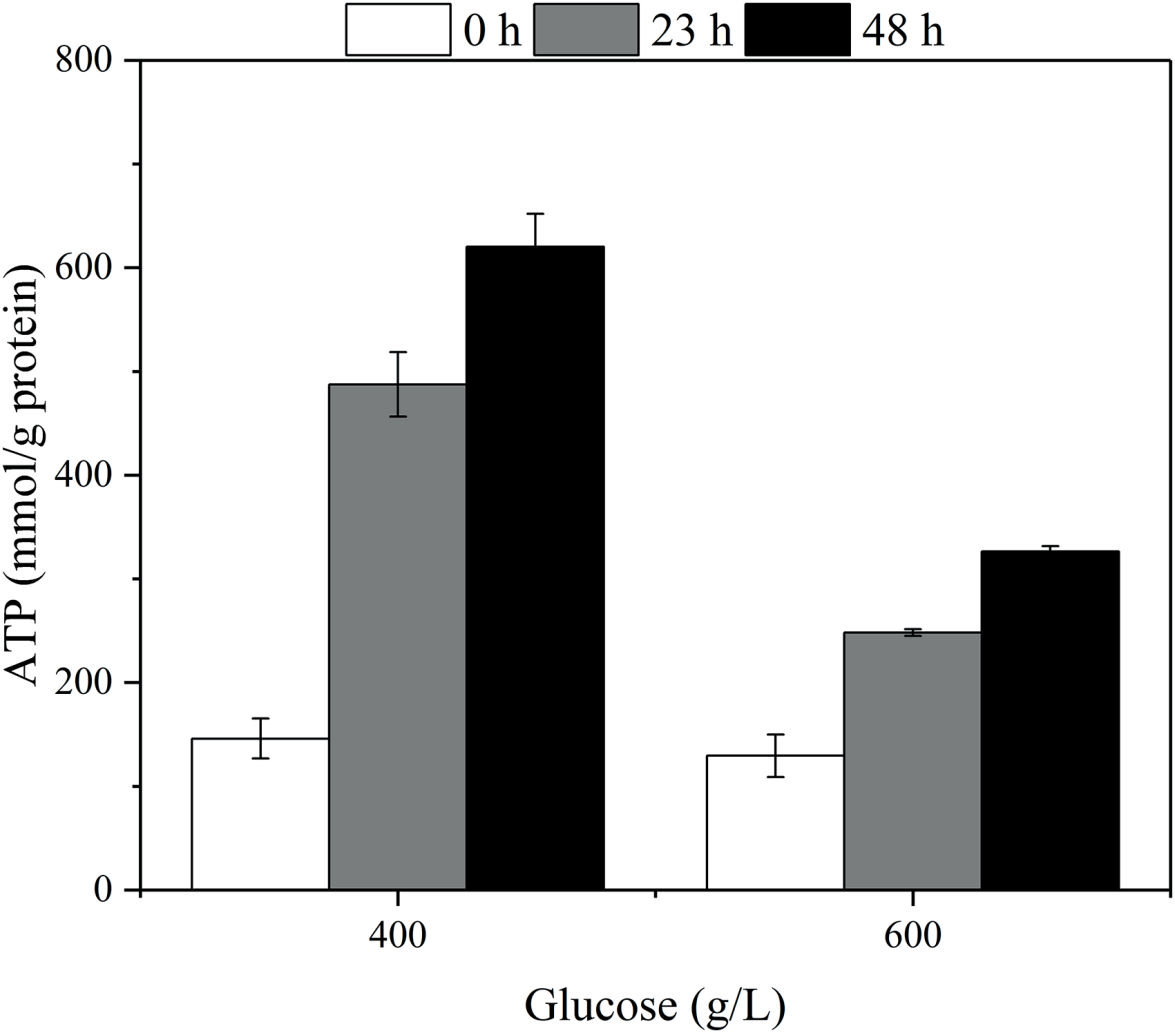
The effect of glucose titers on the intra-cellular ATP concentration of the *G. oxydans*.

The changes in intracellular ATP with different fermentation times were measured at the initial glucose titers of 400 g/L and 600 g/L (Figure 6). The intracellular ATP content was elevated with the increase of fermentation time corresponding to the glucose consumption rate.

### Transcriptome analysis of *Gluconobacter oxydans* to extra-high titers of glucose

**Figure 7:**
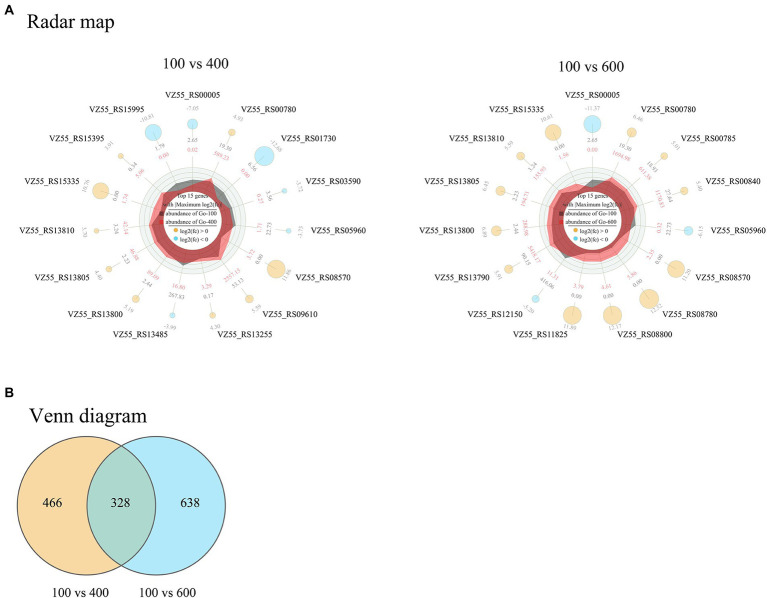
Radar map **(A)** and Venn diagram **(B)** of DEGs in *G. oxydans* responding to extra-high titers of glucose (400 and 600 g/L) with 100 g/L as control. The filter criteria of DEGs were *q* < 0.05 and |log_2_FC| > 1. A parallel experiment was conducted in triplicate. **(A)** The outermost circle are the gene name and log_2_FC; the yellow and sky-blue circle represent up-regulated and down-regulated genes, respectively, and the size of the circle represents the size of the log_2_FC value; outer data of third circle represents the average expression amount of the initial 100 g/L glucose and inner data of third circle represents the average expression amount of the initial 400 g/L (or 600 g/L) glucose; the irregular shapes in the circles are the expression abundance on each axis for initial 100 g/L glucose and initial 400 g/L (or 600 g/L) glucose; the innermost circle center indicates the legend.

In combination with the effects of different initial glucose titers on cellular metabolism, fermentation, growth, and survival of *G. oxydans*, the samples treated with 100 (control), 400, and 600 g/L glucose for 8 h were subjected to transcriptome analysis. A total of 1,432 differentially expressed genes (DEGs) corresponding to high osmotic pressure were obtained (Figure 7B). These two groups showed consistent trends but different specific fold changes in 6 of the top 15 genes with |Maximum log_2_FC|. While the remaining 9 genes were unique DEGs to each group (Figure 7A). These indicated that *G. oxydans* had a different response to extra-high titers of glucose and there were significant differences between groups.

**Figure 8:**
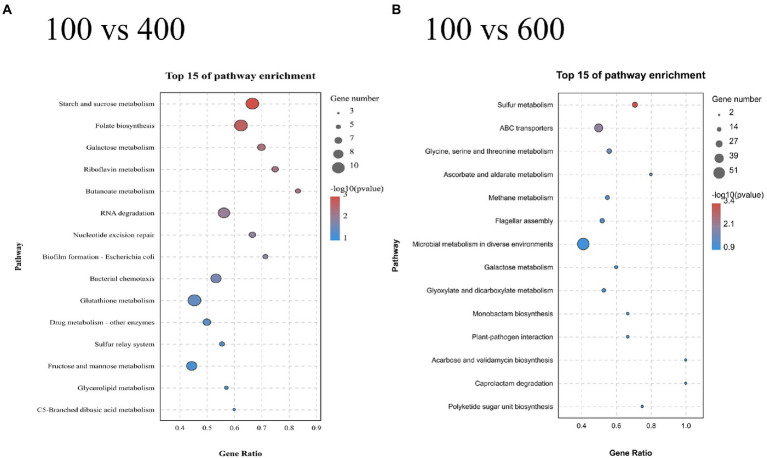
KEGG enrichment analysis of the DEGs obtained at 400 g/L **(A)** and 600 g/L **(B)** glucose with 100 g/L as control. A parallel experiment was conducted in triplicate.

The results of KEGG enrichment of 400 and 600 g/L glucose-treated samples are illustrated in Figure 8. The samples treated with 600 g/L glucose were significantly enriched in the sulfur metabolism pathway (Figure 8B). Besides, 2 DEGs (VZ55_RS10120, VZ55_RS03000) associated with arginine biosynthesis were found to be significantly up-regulated. The samples treated with 400 g/L glucose were significantly enriched in the starch and sucrose metabolism pathway (Figure 8A). Ten DEGs were attributed to this pathway, including 3 genes associated with sucrose metabolism (VZ55_RS06725, VZ55_RS11225, and VZ55_RS04715) and two genes associated with trehalose (VZ55_RS05465 and VZ55_ RS05470).

### RNA-Seq data validation by qRT-PCR

**Figure 9:**
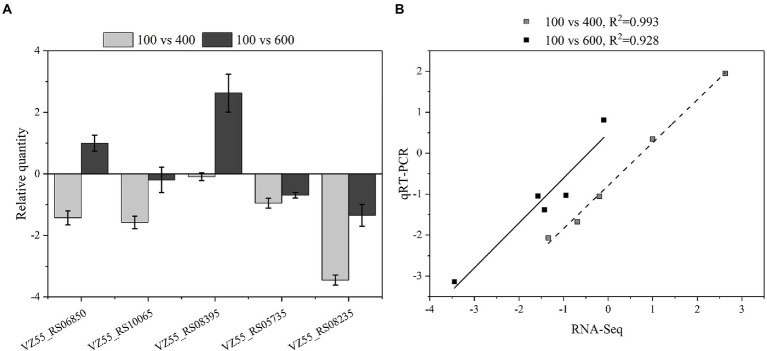
Compared transcription levels of genes of interest by qPCR and RNA-Seq. **(A)** Gene expression levels in qPCR. **(B)** Comparison of gene expression level between qPCR and RNA-Seq. A parallel experiment was conducted in triplicate.

Five DEGs (VZ55_RS10065, VZ55_RS08235, VZ55_RS13980, VZ55_RS08395, VZ55_RS09870) about glucose metabolism on the periplasmic space, including both up-regulated and down-regulated genes, were selected. The transcript levels of *G. oxydans* treated with different titers of glucose (100, 400, and 600 g/L) for 8 h were analyzed by qRT-PCR to verify the reliability of the RNA-Seq analysis results. As illustrated in Figure 9A, the relative expression of the five genes tested showed two patterns of down-regulation followed by up-regulation and always down-regulation upon osmotic stress attack. This result suggested that *G. oxydans* antagonized different high titers of glucose in different ways, and adopted a variety of strategies to antagonize osmotic stress. Although the gene expression fold changes for the five DEGs detected by qRT-PCR were slightly lower than the RNA-Seq data, the trends in expression multiples were consistent with the transcriptome analysis (Figure 9B).

## Discussion

The colony morphological traits alterations could be a macroscopic manifestation of several biological strategies adopted by microorganisms to resist stress conditions, such as starvation, antimicrobial resistance, and osmotic pressure (Sousa et al., 2013; Wang et al., 2019). Compared with liquid medium, bacterial immobilization often inhibits their growth and increases their sensitivity to environmental stress under adverse conditions (Jeanson et al., 2015). Therefore, we specifically compared the growth on agar plate and fermentation behavior in liquid media with typical Gram-negative bacteria (*E. coli*) and eukaryotic strain (*S. cerevisiae*). Both *S. cerevisiae* and *G. oxydans* showed adaptation behavior in the medium of initial 400–600 g/L glucose titers, but the adaptation time of *G. oxydans* was longer. The performance of *G. oxydans* in an extreme high titer liquid medium was not inferior to that of *S. cerevisiae*.

Further, we examined the effect of high glucose titers on the specific physiological state of *G. oxydans*. Both the viable count and whole-cell activity continued to decrease in the high glucose titer medium (400–600 g/L). For organic acid fermentation process, the increased environmental osmotic stress always caused by adding a neutralizer (Tian et al., 2014). Thus, the osmotic pressure in the medium increased due to the accumulation of sodium gluconate resulting in a significant decrease of viable count and whole-cell activity. As described previously, *G. oxydans* showed an adaptation behavior to the high osmotic stress generated by high glucose titers (400–600 g/L), and the glucose consumption rate increased after the adaptation phase (Figure 2B). The results were contradictory to each other. *Gluconobacter oxydans* could gain energy from the incomplete oxidation of glucose to GA (Ehrenreich and Liebl, 2017). However, it could not obtain enough energy from the incomplete oxidation process due to the high osmotic stress. Hua et al. reported that when the uncoupling agent (2,4-Dinitrophenol) disrupted the proton gradient resulting in the loss of ATP synthesis driving force, *G. oxydans* promoted catabolic substrates utilization, such as sorbitol and glucose through the substrate level phosphorylation pathway to compensate for the lack of ATP (Hua et al., 2020a). Thus, it was speculated that *G. oxydans* enhanced the substrate level phosphorylation to obtain sufficient energy. The results of ATP content assay confirmed that the intracellular ATP content did increase continuously after adaptation phage indicating that *G. oxydans* gained more energy due to the increased substrate level phosphorylation.

The 17 DEGs concentrated in the sulfur metabolism pathway (600 g/L) were mainly associated with cysteine synthesis. Cysteine synthesis in Gram-negative bacteria involved two main pathways. In the first route, sulfate was converted to 3′-phosphoadenosine-5′-phosphosulfate by sulfate adenylyltransferase, and subsequently reduced to sulfite (Kertesz, 2006). In the second route, extracellular alkanesulfonate was transferred into the cell and oxidized by alkanesulfonate monooxygenase, resulting in the production of aldehyde and sulfite (Park et al., 2020). The sulfite generated by both pathways was reduced to sulfide by sulfite reductase and subsequently transferred onto O-acetylserine to yield cysteine. Four DEGs involved in first route (VZ55_RS06350, VZ55_RS06345, VZ55_RS06340, and VZ55_RS09905), 10 DEGs involved in the second route (VZ55_RS12750, VZ55_RS12775, VZ55_RS12825, VZ55_RS13290, VZ55_RS13365, VZ55_RS12795, VZ55_RS13385, VZ55_RS12790, VZ55_RS13275, and VZ55_RS12785), and 3 DEGs associated with the conversion of sulfite to cysteine (VZ55_RS05185, VZ55_RS10370, and VZ55_RS04470), were all significantly down-regulated. This may be to strictly regulate the cysteine content to control its toxicity to cells (Sorensen and Pedersen, 1991; Takumi et al., 2017). In addition, two arginine-related were upregulated. The intracellular arginine concentration did increase with the initial glucose titers (Supplementary Figure S1). Xu et al. (2011) reported that *Candida glabrata* transported large amounts of arginine from the environment to the cell as a compatible solute to counteract osmolality in the environment. The osmotic tolerance of microorganisms was closely related to the compatible solutes. A study by Liang et al. on freshwater cyanobacterium *Synechococcus elongatus* PCC 7942 found that it exclusively accumulated sucrose as a compatible solute upon salt stress (Liang et al., 2020). Trehalose is a non-reducing disaccharide found in many organisms from bacteria to mammals (Thierry et al., 2011). In bacteria, trehalose could be utilized as a carbon and energy source and accumulated as a compatible solute for its specific physical properties (Vyrides and Stuckey, 2017; Thorwall et al., 2020; Zeidler and Müller, 2020). Studies by Woodcock et al. showed that *Pseudomonas aeruginosa* collaboratively antagonized osmolarity by producing trehalose and α-glucan (Woodcock et al., 2021). Thus, it was speculated that the 5 DEGs enriched to this pathway might be associated with compatible solutes formation. However, no intracellular trehalose was detected by high-performance liquid chromatography (coupled with Aminex HPX-87H). This may be due to the involvement of up-regulated OtsA (VZ55_RS05465) in the regulation of cell morphology (Chen et al., 2017) or glycolysis (Eastmond and Graham, 2003). Although Zahid et al. found that mannitol was a compatible solute against the sucrose mediated osmotic pressure in *G. oxydans*, intracellular mannitol was not found under high glucose titer mediated hypertonic condition.

## Conclusion

In this study, the effects of high titer glucose on fermentation performances and cell morphology of *G. oxydans* were investigated. The results showed that *G. oxydans* could not grow on the 600 g/L glucose agar medium and showed a certain metabolic capacity in the corresponding liquid medium, reaching the highest tolerance ability of *S. cerevisiae*. *Gluconobacter oxydans* obtained efficient energy by enhancing the substrate level phosphorylation, resulting in the increased glucose consumption rate. A total of 1,432 DEGs corresponding to high osmotic stress were obtained from the transcriptome analysis. Several genes related to compatible solutes were founded, primarily associated with trehalose and arginine metabolism.

## Data availability statement

The datasets presented in this study can be found in online repositories. The names of the repository/repositories and accession number(s) can be found below: SRA, PRJNA755830.

## Author contributions

XL: conceptualization, formal analysis, investigation, data curation, and writing—original draft. ZW: investigation. JX: investigation. XZ: writing—review and editing. YX: conceptualization, funding acquisition, supervision, and writing—review and editing. All authors contributed to the article and approved the submitted version.

## Funding

The research was supported by the National Natural Science Foundation of China (32171730) and the Key Research and Development Program of Jiangsu (BE2015758). Also, the authors gratefully acknowledge financial support from the Priority Academic Program Development of Jiangsu Higher Education Institutions.

## Conflict of interest

The authors declare that the research was conducted in the absence of any commercial or financial relationships that could be construed as a potential conflict of interest.

## Publisher’s note

All claims expressed in this article are solely those of the authors and do not necessarily represent those of their affiliated organizations, or those of the publisher, the editors and the reviewers. Any product that may be evaluated in this article, or claim that may be made by its manufacturer, is not guaranteed or endorsed by the publisher.

## Supplementary material

The Supplementary material for this article can be found online at: https://www.frontiersin.org/articles/10.3389/fmicb.2022.977024/full#supplementary-material
