# Supplementary material for: Osmotic stress tolerance and transcriptome analysis of Gluconobacter oxydans to extra-high titers of glucose
Source: Front Microbiol. 2022 Aug 12;13:977024. doi: 10.3389/fmicb.2022.977024 (PMC9412170; doi:10.3389/fmicb.2022.977024)
Supplement: Supplementary file 1 [file Data_Sheet_1.docx]

Supplementary Material

## 1. Supplementary Figure





**Figure S1.** The effect of glucose titers on the intra-cellular arginine concentration of the *G. oxydans*.
